# The Poor Man's Home.—XX

**Published:** 1898-01-08

**Authors:** 


### MODERN SOCIOLOGY.

### THE POOR MAN'S HOME.—XX.

### HOUSING IN GERMANY (*continued*).

EVERY town in Germany seems to have some distinctive feature in its method of house-building. Herr Meyer's tenements and others in Leipsic which we have not described, such as Mrs. Emma Hasse's "Goldnen Hohe" and the dwellings erected by the Salomon Fund, have all the feature of the spare ground being cut up into small gardens. This is a very admirable idea, for it gives the men who live there a wholesome interest to occupy their spare time. When we hear of picnics being organised to take place in the microscopic gardens of the Meyer tenements, we cannot help being amused at the idea; it seems to partake so much of that habit of make-believe which with us is confined to children, but which foreigners seem to preserve all their lives. It is a harmless trick of the imagination, and helps to make life pleasanter; so perhaps we do ill to smile at it.

These gardens, as we have said, are characteristic of Leipsic, while in Dresden many tenements are built in double blocks, back to back, but, we hasten to add, with a court between. In this court are the drying-ground and the playground, and also out-buildings of different kinds, such as laundries, coal-cellars, and the like. Corridors connect the front and rear parts of the blocks, and in these the privies are placed, which are thus easily arranged to be well lighted and ventilated. This is the more important as they are all dry, and the pit into which the refuse falls is not emptied more than once a month.

The tenements erected by the Co-operative Building Society are good specimens of the Dresden style. The front and back blocks are separated by a space of about 25 ft., and the corridors that cross from one part of the building to the other are 20 ft. wide. The staircases are practically outside the main building, so that the whole of the block is given up to the lodgings. These, as built, are intended as to be three and four room houses. Each has a private hall within its door. This arrangement is convenient in many ways. As each door of the dwelling opens on to this hall, it is possible to divide a tenement which is too large for one family into two separate lodgings. Where one family occupies the whole tenement the hall is often used as a kitchen, for the arrangement of the flues permits this, and the kitchen can be kept for other purposes. The sink is in this private hall, so that it always serves the purpose of a scullery. There is an unlimited supply of water in the buildings, an advantage not so frequently found on the Continent as in England. A cooking-range is provided, and china stoves for heating in the larger rooms, which can also be adapted for cooking purposes. There is gas in the corridors, but oil is used for private lighting in the rooms.

In the three-room tenements the kitchen and the private hall are about the same size, viz., 11½ ft. long by 7½ ft. wide. There is also a living-room which is 14½ ft. long and 12½ ft. wide, and a bedroom, the length of which is the same as the living-room, while its width is 7 ft. 10 in. In the four-room lodgings the kitchen is generally smaller than in the three-room flats, the size of the other rooms being the same. The enterprise is conducted by a co-operative building association, which, however, forbids the paying of dividends beyond 4½ per cent. Hitherto only 4 per cent. has been paid. So far, all the tenants have been shareholders, but as the work goes on the association may let houses to outsiders. The method by which rents are reckoned is rather original. It is calculated on a basis of 5d. per square foot of space (including walls and partitions) per annum for the ground floor, and the same for the fourth. The first and second floors, which are considered to be more desirable are charged 10 per cent. more, and the third 5 per cent., while for the top storey a deduction of 15 per cent. is made. At this rate the rental of three room flats on the ground floor is 200 marks (£9 18s. 4d.), the second floor ones about £1 dearer, and those on the fifth floor about £2 cheaper. The cheapest four-room tenements cost about £10, and the dearest 302 marks, or within a few pence of £15. Tenants who find their house too large for their own needs are permitted to sublet rooms to single persons, but such rooms must have a separate entrance from the hall. One tenant is chosen as janitor. His task is to keep order and to attend to the lighting of the stairs. His remuneration amounts only to 20 marks (£1) a year, but it is considered an honour to obtain the post, and as the tenants are themselves part owners of the property, they take better care of the buildings than ordinary tenants would, so the matter of keeping order is not a very onerous task. The janitor has also charge of the laundry, for the use of which tenants pay 1¼d. every time. They are allowed to avail themselves of it only one day a month. Those who wish to wash more frequently than this, or who take in washing, are allowed to do so in an out-house, for the use of which a small extra fee is charged. The model tenements of the St. John's Society in Dresden resemble in most respects those of the Co-operative Society, which we have described.

In the town of Hanover the "Savings and Building Society" has a block of tenements, which, although not quite so good as those we have spoken of in Dresden and Leipsic, seem to be much appreciated. Every vacant tenement is applied for at least five times over. While the rooms are much the same as those in the houses we have spoken of, there is the disadvantage that each family has not a privy for its own use (of course, these are dry, as in the other towns), and there is no special laundry provided, though washing may be done in the cellar. The rent in these Hanover houses also takes a larger proportion of the family income than in the other places we have dealt with, about 20 to 25 per cent. being the average. Yet similar accommodation in the neighbourhood would cost about a third more. Although this society has a few three-room flats, the majority of their dwellings are of four rooms One of the latter contains a kitchen measuring 11 ft. 10 in. by 8½ ft., a living-room of good size—13 ft. 9 in by 12½ ft.—and two bedrooms, 14 ft. 9 in. long, and 9 ft. 6 in. wide. There is also a private hallway 8½ ft. in length and 4 ft. 9 in. wide. Such a house rents for 180 marks on the highest and lowest storeys, for 200 marks on the second, and for 190 on the third (£8 18s. 6d., £9 18s. 4d., and £9 8s. 2½d. respectively).

